# Extracellular vesicles are associated with C-reactive protein in sepsis

**DOI:** 10.1038/s41598-021-86489-4

**Published:** 2021-03-26

**Authors:** Birgit Fendl, René Weiss, Tanja Eichhorn, Ingrid Linsberger, Taras Afonyushkin, Florian Puhm, Christoph J. Binder, Michael B. Fischer, Viktoria Weber

**Affiliations:** 1 Christian Doppler Laboratory for Innovative Therapy Approaches in Sepsis, Department for Biomedical Research, Danube University Krems, Dr.-Karl-Dorrek-Strasse 30, 3500 Krems, Austria; 2 Department of Laboratory Medicine, Medical University of Vienna, Vienna, Austria; 3 Center for Experimental Medicine, Department for Biomedical Research, Danube University Krems, Krems, Austria

**Keywords:** Inflammation, Sepsis, Immunology, Medical research

## Abstract

There is increasing evidence that C-reactive protein (CRP) can mediate inflammatory reactions following the transformation of functionally inert pentameric CRP (pCRP) into its structural isoform pCRP* and into monomeric CRP (mCRP). This conversion can occur on the membranes of apoptotic or activated cells or on extracellular vesicles (EVs) shed from the cell surface. Here, we characterized the association of CRP with EVs in plasma from sepsis patients using flow cytometry, and found highly elevated levels of total EV counts and CRP^+^ EVs as compared to healthy individuals. We further assessed the ability of PentraSorb CRP, an extracorporeal device for the adsorption of CRP, to deplete free CRP and CRP^+^ EVs. Treatment of septic plasma with the adsorbent in vitro resulted in almost complete removal of both, free CRP and CRP^+^ EVs, while total EV counts remained largely unaffected, indicating the detachment of CRP from the EV surface. EVs from septic plasma elicited a release of interleukin-8 from cultured human monocytes, which was significantly reduced by adsorbent treatment prior to EV isolation. Our findings provide evidence that CRP^+^ EVs exhibit pro-inflammatory characteristics and can contribute to the spreading of inflammation throughout the circulation on top of their pro-coagulant activity.

## Introduction

C-reactive protein (CRP) is an acute phase protein that is mainly produced by hepatocytes in response to tissue injury, inflammation, and sepsis^1,2^, where CRP levels can rise from 1 mg/L up to 500 mg/L within 24–72 h^3^. Moreover, strongly elevated CRP plasma levels have recently been shown to correlate with disease severity and prognosis in COVID-19 pneumonia^4^. In plasma, CRP is present as a disc-shaped homopentamer (pCRP), which dissociates into its monomers (mCRP) upon tissue deposition^5^, leading to the exposure of distinct neo-epitopes^6^. The dissociation is mediated by exposure of CRP to lysophosphatidylcholine, a bioactive lipid generated following phospholipase A2 expression on activated cell membranes^7^.

pCRP possesses an effector and a binding face^8,9^. The binding face attaches to apoptotic or necrotic cells via oxidized phosphatidylcholine residues that expose phosphocholine on cell membranes^10,11^, whereas the effector face mediates CRP binding to cell-bound Fcγ receptors^5,9,12^ and to C1q^8,13^. While pCRP exerts anti-inflammatory characteristics in the circulation^13^, tissue-bound mCRP induces the upregulation of cell adhesion molecules on neutrophils, monocytes, and endothelial cells, and triggers complement activation. Only recently, extracellular vesicles (EVs) have been found to bind and to transfer mCRP to activated endothelial cells, contributing to the dissemination of inflammation^14^.

EVs are sub-cellular fragments originating from the endosomal system or shed from the plasma membrane of virtually all human cell types under both, physiological or pathological conditions^15^. They are key players in intercellular communication and exhibit vital roles in hemostasis, angiogenesis, inflammation, immune response, and waste management^16–18^. In sepsis, EVs can support inflammation by increasing cytokine production and cell surface molecule expression on endothelial cells and leukocytes^19,20^, but can also reduce chemotaxis and phagocytic activity of immune cells^21^. They promote coagulation via the exposure of phosphatidylserine and tissue factor^22,23^ and can contribute to the development of disseminated intravascular coagulation^24^.

There is abundant evidence that CRP is associated with circulating EVs in patients suffering from rheumatoid arthritis^25,26^, myocardial infarction^27^, and peripheral arterial disease^28^. EV-bound CRP (pCRP*) undergoes a conformational re-arrangement on the EV surface and may ultimately dissociate into its monomeric isoforms. Both, pCRP* and mCRP exhibit pro-inflammatory characteristics^8^, and CRP is therefore increasingly considered as a contributor to tissue injury in various diseases^5,29^.

Two approaches to limit CRP-mediated tissue damage are currently under investigation. Next to the specific small-molecule CRP inhibitor 1,6-bis(phosphocholine)-hexane, which occludes the binding face of pCRP and thereby blocks its functions^30^, an extracorporeal device to deplete CRP from human plasma by adsorption to beads functionalized with phosphocholine (PentraSorb CRP) has been introduced. It is intended for application in patients suffering from acute myocardial infarction or from other acute inflammatory disorders with elevated CRP plasma levels^31–33^.

In the present study, we assessed the association of CRP with EVs in plasma from sepsis patients and tested the capacity of PentraSorb to deplete CRP-carrying EVs from septic plasma in vitro. Our findings confirm a high degree of interaction of EVs with CRP in sepsis patients and provide evidence for the efficient depletion of both, soluble and EV-associated CRP using PentraSorb.

## Results

### Plasma from sepsis patients contains elevated levels of CRP and CRP^+^ extracellular vesicles

**Figure 1:**
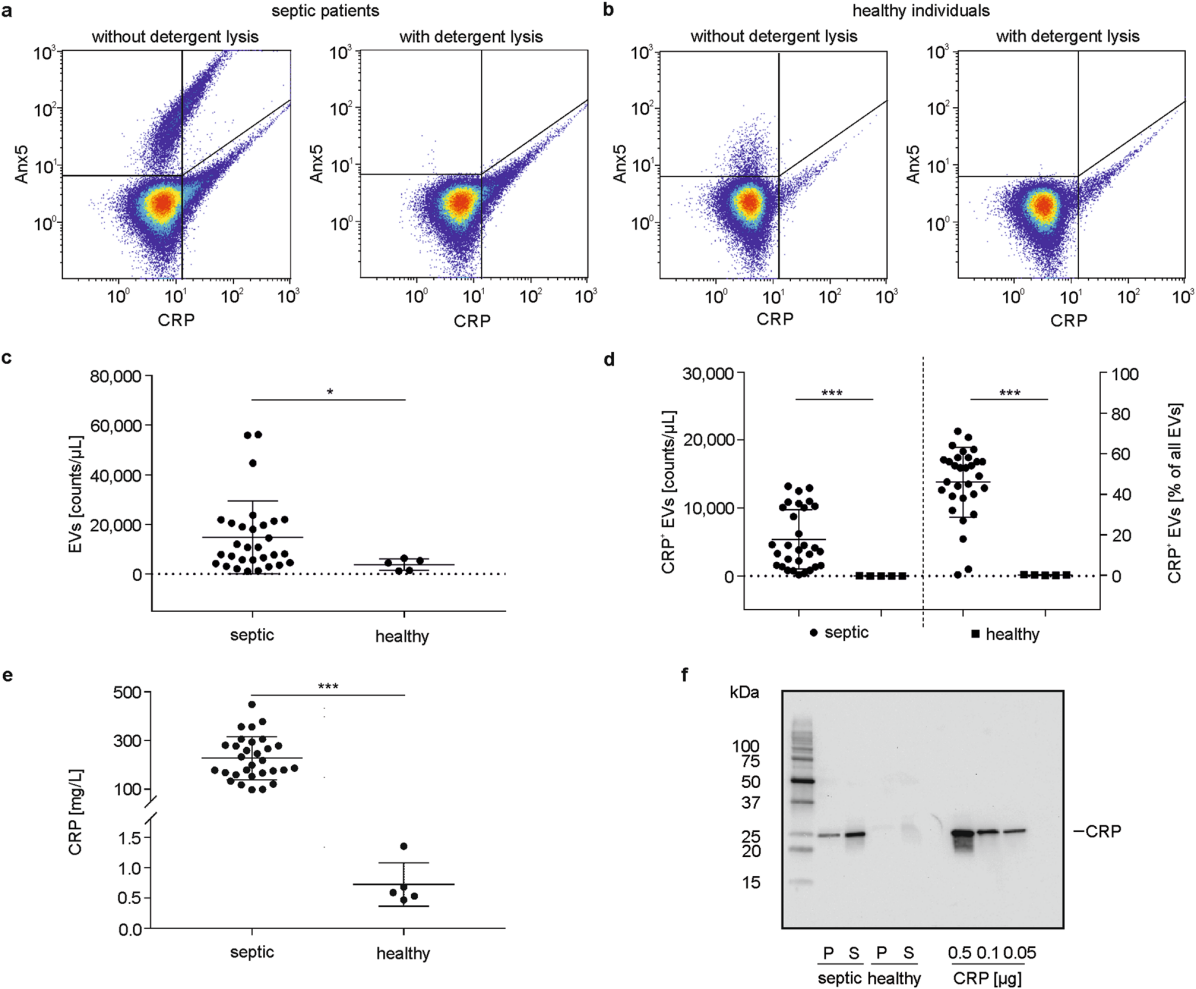
Characterization of plasma samples from sepsis patients and healthy individuals. **(a)** Plasma from sepsis patients and **(b)** plasma from healthy individuals was stained with anti-CRP-FITC and Anx5-PE to identify CRP-carrying EVs (CRP^+^ EVs) using flow cytometry. Detergent lysis with Triton X-100 abolished the EV cloud, confirming the presence of intact vesicles. Total EV counts **(c)**, and the association of EVs with CRP **(d)** were determined in plasma from sepsis patients (n = 30) as compared to healthy individuals (n = 5); **(e)** Levels of plasma CRP in sepsis patients *vs.* healthy individuals. Data are given as mean ± SD and were compared using a Mann–Whitney test; *p < 0.05, ***p < 0.001. **(f)** EVs were pelleted from plasma by centrifugation and both EV-depleted supernatant and the EV pellet (15 µg protein each) were separated by SDS-PAGE under reducing conditions and probed for CRP by Western blotting. Human CRP was used as positive control (0.5, 0.1, 0.05 µg).

For the flow cytometric identification of pro-coagulant EVs, we relied on their exposure of phosphatidylserine, and defined EVs as Annexin5-positive (Anx5^+^) events in the EV gate (Supplementary Fig. S1a). Pre-treatment of plasma samples with Triton X-100 abolished the Anx5^+^ EV cloud both in plasma from sepsis patients and from healthy individuals (Fig. 1a,b), proving the presence of intact detergent-sensitive vesicles^34^.

Septic plasma contained higher levels of EVs as compared to healthy individuals (14,732 ± 14,657 EVs/µL *vs.* 3741 ± 2328 EVs/µL; n = 30 for sepsis patients, n = 5 for healthy individuals; Fig. 1c). The majority of EVs originated from platelets, as shown by staining with anti-CD41-PC7 (65.2 ± 6.8% CD41^+^ EVs of all Anx5^+^ EVs, n = 3). In septic plasma, 45.9 ± 17.2% of all EVs were associated with CRP (5359 ± 4364 CRP^+^ EVs/µL; n = 30), while we failed to detect significant amounts of CRP^+^ EVs in healthy individuals (0.2 ± 0.2% of all EVs; 6 ± 8 CRP^+^ EVs/µL; n = 5; Fig. 1d). Again, the majority of CRP^+^ EVs were platelet-derived (70.9 ± 12.8% CD41^+^ EVs of all CRP^+^Anx5^+^ EVs; n = 3).

Plasma CRP levels were 227.0 ± 88.6 mg/L for sepsis patients (n = 30) *vs.* 0.7 ± 0.4 mg/L for healthy individuals (n = 5; Fig. 1e). Notably, plasma CRP levels were not correlated with CRP^+^ EVs in plasma from sepsis patients.

Western blotting confirmed the presence of CRP in EV fractions enriched by centrifugation of septic plasma and in the respective supernatants, while CRP remained undetectable in samples from healthy individuals (Fig. 1f).

### Plasma CRP is efficiently depleted by treatment with PentraSorb

**Figure 2:**
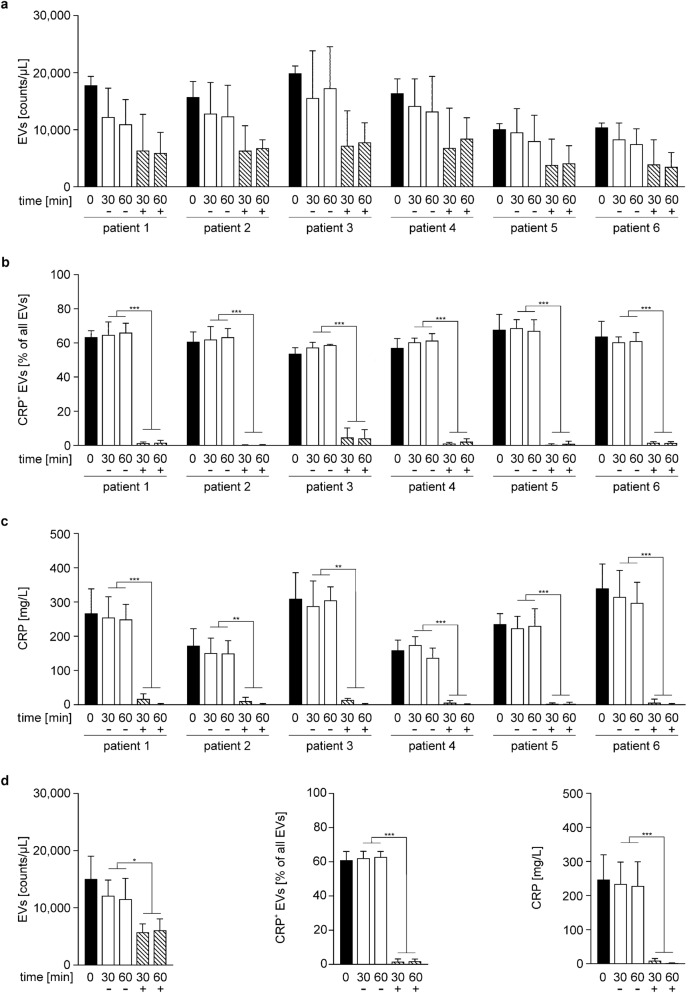
Depletion of CRP and CRP^+^ extracellular vesicles from plasma from sepsis patients. Plasma from sepsis patients (6 patients; n = 3 for each patient) was incubated with (+) or without (−) PentraSorb for 30 and 60 min to deplete CRP, as described in the Methods section. EV counts **(a)**, CRP-carrying (CRP^+^) EVs **(b)**, as well as plasma CRP **(c)** were quantified at baseline (0 min), and after 30 and 60 min. The decrease of plasma EV counts over time was mainly due to unspecific binding of EVs by the adsorbent, as a comparable drop in EV counts was observed for the non-functionalized adsorbent matrix (non-functionalized agarose), as shown in Supplementary Fig. S2. CRP^+^ EVs and plasma CRP were efficiently depleted by PentraSorb treatment. **(d)** Summary of data as mean of all six patients for EV counts, CRP^+^ EVs, as well as plasma CRP. Data are given as mean ± SD and were compared using a two-way ANOVA; *p < 0.05, **p < 0.01, ***p < 0.001.

To determine the ability of the CRP adsorbent PentraSorb to deplete soluble CRP as well as CRP^+^ EVs in vitro, we selected plasma samples from specific sepsis patients that contained at least 50% of CRP^+^ EVs (n = 6). The overall EV counts in plasma decreased slightly during incubation with the adsorbent (baseline: 15,053 ± 3992 EVs/µL; 60 min: 11,545 ± 3628 EVs/µL without adsorbent *vs.* 6097 ± 1973 EVs/µL with adsorbent; n = 6, mean of all six patients; Fig. 2a,d). We suggest that this decrease was rather due to unspecific interaction of EVs with the adsorbent beads than to specific binding, as incubation with non-functionalized agarose (adsorbent matrix) resulted in a comparable decrease in EV counts (Supplementary Fig. S2a). CRP^+^ EVs, however, were barely detectable after adsorbent treatment (60 min: 1.8 ± 1.3% CRP^+^ EVs), while their levels remained unaffected in untreated plasma (baseline: 61.0 ± 5.0% CRP^+^ EVs; 60 min: 62.9 ± 3.2% CRP^+^ EVs; n = 6; Fig. 2b,d). This indicates that CRP was detached from EVs by PentraSorb treatment, while the percentage of CRP^+^ EVs remained unaffected by treatment with non-functionalized agarose (Supplementary Fig. S2b).

Plasma CRP was efficiently depleted by PentraSorb treatment (baseline: 247.2 ± 72.6 mg/L CRP; 60 min: 228.1 ± 71.4 mg/L CRP without adsorbent *vs.* 1.8 ± 0.7 mg/L CRP with adsorbent; n = 6; Fig. 2c,d).

### CRP^+^ extracellular vesicles induce IL-8 secretion in human monocytes

**Figure 3:**
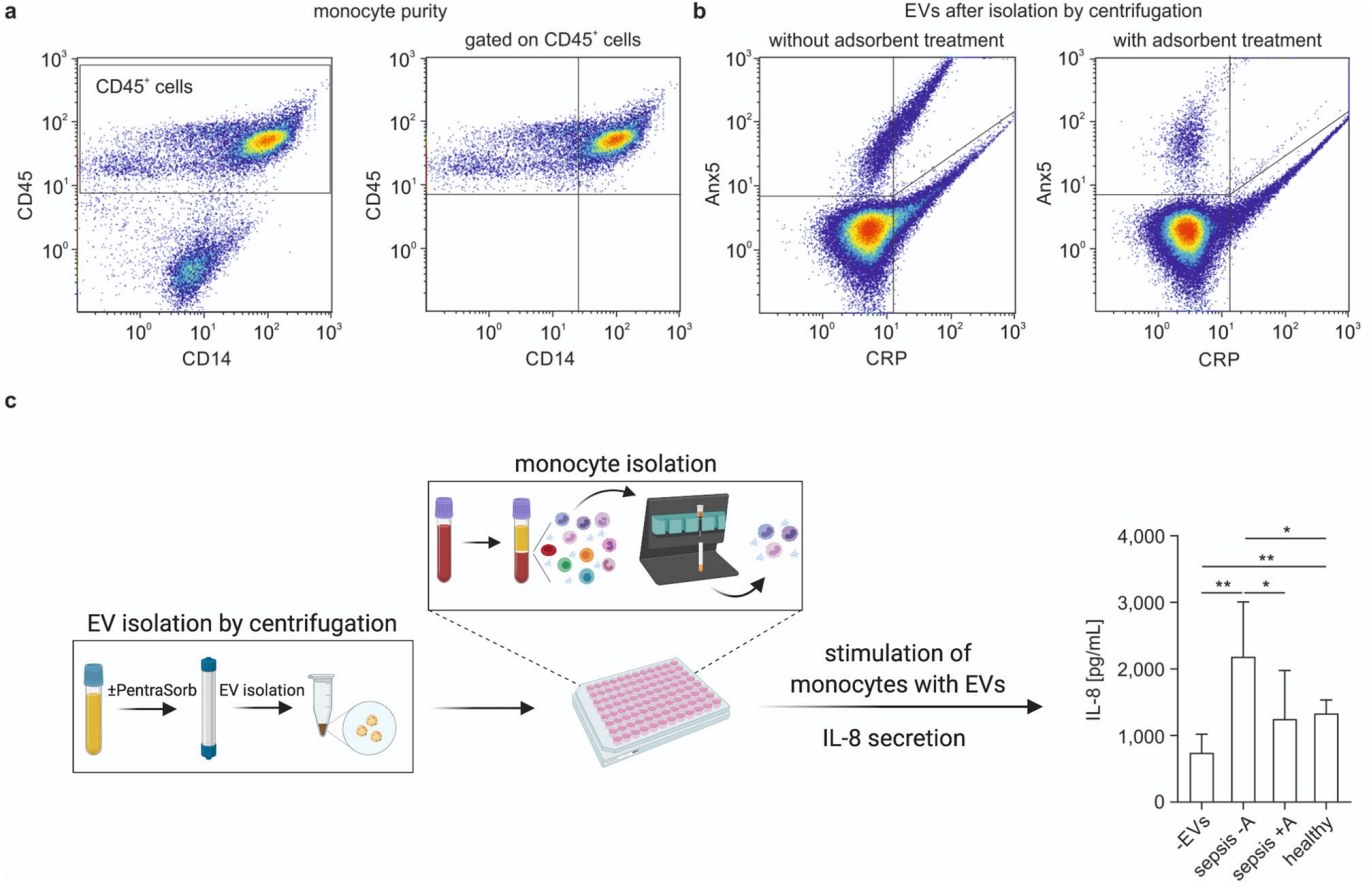
Stimulation of monocytes with extracellular vesicles. **(a)** Human primary monocytes were isolated from peripheral blood mononuclear cells by negative depletion, as described in the Methods section. Cells were stained with anti-CD14-PE and anti-CD45-PB, monocytes were identified as CD45^+^CD14^+^ cells, and their purity was calculated in relation to all leukocytes (CD45^+^ cells). **(b)** EVs were enriched by centrifugation from plasma from septic patients without (left) or with (right) PentraSorb treatment. **(c)** Monocytes were stimulated with EVs from septic plasma, which was either pre-treated with PentraSorb (sepsis + A) or left untreated (sepsis -A). IL-8 was quantified in the supernatant by ELISA. Incubation of monocytes without EVs (-EVs) or with EVs from healthy individuals (healthy) served as control. n = 5 (-EVs); n = 7 (sepsis -A); n = 7 (sepsis + A); n = 4 (healthy). Data were compared using an unpaired t-test with or without Welch correction. Data are given as mean ± SD; *p < 0.05, **p < 0.01. Part **(c)** created with *BioRender.com* (Academic Subscription).

To evaluate the biological activity of CRP^+^ EVs, we characterized interleukin 8 (IL-8) secretion by human monocytes upon stimulation with EVs from septic plasma. Isolated primary human monocytes exhibited a purity of 71.0 ± 4.4% (Fig. 3a and Supplementary Fig. S3) and a viability of 93.0 ± 1.7%. Stimulation of monocytes with EVs enriched from septic plasma (Fig. 3b) induced a significant release of IL8 (2185.0 ± 822.2 pg/mL, n = 5; unstimulated control 741.6 ± 279.4 pg/mL, n = 7; Fig. 3c). Treatment of septic plasma with PentraSorb prior to EV enrichment effected a significantly reduced IL-8 release from monocytes (1250.0 ± 728.3 pg/mL; n = 7) with IL-8 levels that were comparable to those observed for monocyte stimulation with EVs from healthy individuals (1333.0 ± 202.9 pg/mL; n = 4).

## Discussion

The pro-coagulant activity of EVs is mainly based on their exposure of phosphatidylserine, which catalyzes the assembly of coagulation complexes on the EV surface^35^. Here, we characterized the interaction of pro-coagulant EVs from sepsis patients with CRP, which, apart from being a marker of inflammation, can actively mediate tissue damage following structural changes from its native, pentameric state into its monomeric form. As these conformational changes can occur on activated platelets, apoptotic cells, or upon binding of CRP to EVs, CRP-carrying EVs are likely to support both, coagulation and inflammation, making them interesting targets in inflammatory disorders, such as sepsis^8,9^.

In our study, we used flow cytometry to characterize EVs directly in plasma. This approach allows for the detection and quantification of EVs as well as for the identification of their cellular origin and their association with plasma proteins, without requiring additional pre-analytical sample preparation steps^36^. We identified pro-coagulant EVs by binding of fluorescently labeled Anx5 to phosphatidylserine exposed on the EV surface and included CD41 as a marker for platelet-derived EVs, which constitute the majority of circulating EVs. While flow cytometry precludes the analysis of smaller EVs (exosomes) due to its detection limit of approximately 250 nm, this did not constitute a major limitation of our study, as phosphatidylserine is primarily exposed on larger EVs derived from the plasma membrane (microvesicles), so that our target population of CRP^+^Anx5^+^ EVs was well accessible for analysis by flow cytometry.

We found significantly elevated levels of both, total EVs (Anx5^+^) and EVs carrying CRP (CRP^+^Anx5^+^) in sepsis patients as compared to healthy individuals, where CRP^+^ EVs remained undetectable. CRP^+^ EVs were present over the entire size range of the EV population (0.3–1 µm), suggesting that CRP^+^ events were not, or at least not predominantly, derived from CRP-decorated apoptotic bodies (size range 1–5 µm)^37^. Furthermore, detergent lysis completely abrogated CRP^+^ events in the EV gate, supporting the presence of intact CRP^+^ vesicles. Notably, we observed two subpopulations in our patient cohort, which were discriminated by the percentage of CRP^+^ EVs, with two clusters of samples containing an average of 33% and 60% CRP^+^ EVs, respectively. This provides evidence that only certain EV subpopulations are capable of binding to CRP and was further supported by the fact that less than 50% of all Anx5^+^ EVs were associated with CRP across the entire study population despite an extreme excess of plasma CRP.

Contrary to previous studies^27^, we failed to detect a correlation between CRP plasma levels and CRP^+^ EVs in septic plasma. It is, however, questionable whether calculating such a correlation would be permissible at all, since the depletion of CRP^+^ EVs from plasma did not measurably change the overall plasma CRP concentration, indicating that the amount of EV-bound CRP represents only a very small proportion of total plasma CRP. To address this question in more detail, we tried to estimate the amount of CRP that is actually bound to EVs. We based our estimation on the assumption that a minimum of 1000 molecules of fluorescently labeled Anx5 are required to provide a sufficient signal for the detection of a single EV in flow cytometry^38^. Assuming that the detection of a single CRP^+^ EV would require an equal amount of labeled antibody, we calculated that in sepsis patients with average plasma CRP levels of approximately 230 mg/L and 5400 CRP^+^ EVs/µL, the total amount of EV-bound CRP would be equal to 5.6 µg. This corresponds to 0.0005% of total CRP, which immediately raises the question of the biological relevance of EV-bound CRP. This relevance becomes evident, however, in the light of recent advances in our understanding of CRP in inflammation. There is increasing evidence that EVs derived from activated platelets are capable of converting pCRP into pro-inflammatory mCRP. Moreover, EVs can transport mCRP, which is not able to circulate freely due to its poor solubility, and transfer it e.g. to activated endothelial cells^14^. CRP^+^ EVs can therefore play a significant role in disseminating inflammation throughout the circulation^9,14,28^. This might be particularly relevant in settings associated with increased release of EVs, e.g. from activated platelets.

Several lines of evidence support the hypothesis that the binding of CRP to EVs occurs via oxidation-specific epitopes (oxidized phosphatidylcholine) on the EV surface. First, the same Ca^2+-^dependent mechanism is known to mediate the binding of CRP to apoptotic cells and to oxidized LDL^10^ and second, oxidized phosphatidylcholine has also been detected on a subpopulation of circulating EVs from healthy individuals using the natural antibody T15/E06^11,39^. It was therefore appealing to assume that oxidized phosphatidylcholine might also be present on EVs in septic plasma and might act as binding site for CRP. We were, however, unable to confirm the presence of oxidized phosphatidylcholine on EVs in septic plasma samples after staining with T15/E06, which might indicate that all oxidation-specific epitopes on the EV surface were occupied by CRP and were not accessible to the antibody. Alternatively, the binding of CRP to EVs could involve a different, calcium-independent mechanism, as we observed that the interaction of EVs and CRP was not reversed in plasma anticoagulated with ethylene diamine tetraacetic acid (EDTA). It is conceivable that binding of CRP to EVs occurs via an interaction of the CRP effector face with Fcγ receptors^5,9,12^ on the EV surface, as previously shown for CRP binding to endothelial cells^40^. A receptor-mediated binding mechanism would also be compatible with our aforementioned observation that less than 50% of all EVs were associated with CRP even in the presence of excess plasma CRP.

We have previously shown that EVs interact preferentially with monocytes in the circulation^36,41–43^, and stimulation of monocytes with CRP has been found to induce a dose-dependent release of IL-8^44^. Here, we found that incubation of primary human monocytes with CRP^+^ EVs enriched from septic plasma resulted in significantly increased IL-8 release, which was abrogated when septic plasma was treated with the CRP-adsorbent PentraSorb prior to the enrichment of EVs, as discussed below. In our experimental set-up, monocyte isolation was followed by an overnight resting phase, which was associated with a pronounced shift from CD14^++^CD16^-^ classical to CD14^++^CD16^+^ intermediate monocytes, i.e. with an up-regulation of CD16 (FcγRIII) on monocytes, as previously reported^42^. In this previous study, we identified CD16^+^ monocytes as main binding partners of circulating EVs. Since recent findings indicate that CRP is capable of associating with all three Fcγ receptors, but that mCRP exhibits the highest affinity towards FcγRIII/CD16^3^, this might be a hint for an involvement of FcγRIII/CD16 in the binding of mCRP to EVs.

To limit the volume of blood drawn from critically ill patients, we used monocytes from healthy individuals in our study, which were enriched in CD16^+^ monocyte subsets following an overnight resting phase^42^. While this upregulation of CD16 could be interpreted as monocyte maturation towards macrophages, a phenotypic characterization of the isolated monocytes based on their expression of CD14, CD16, CCR2, CX3CR1, and CCR5 strongly suggested the presence of intermediate monocytes rather than a terminal differentiation, as previously described^43^. As discussed in detail in this previous study, residual platelets contained in isolated monocyte preparations might induce a shift towards an intermediate monocyte phenotype. This is actually confirmed by findings by Passacquale et al.^45^, showing that platelets as well as platelet supernatants containing platelet-derived EVs induce a shift towards intermediate monocytes.

Since alterations in monocyte subset distribution have also been described in sepsis, where CD16^+^ intermediate monocytes represent the main subset^46,47^, it is conceivable that CRP^+^ EVs bind to and activate CD14^++^CD16^+^ intermediate monocytes as well as CD14^+^CD16^+^ non-classical monocytes via CD16 under septic conditions^48^. In this context, it is interesting to note that EVs from stroke patients were shown to induce the expression of TNF-α, IL-1β, CXCL-1 and CCL-2 in human THP-1-derived macrophages in vitro^49^. A direct association of CRP with EVs was not analyzed in this study; however, the authors reported a significant correlation between EV counts and CRP, which makes the presence of CRP^+^ EVs in their samples very likely.

As a limitation of our present work, the sample volume remaining after monocyte stimulation did not allow for the quantification of further cytokines or chemokines. This prohibits further conclusions regarding inflammatory effects of CRP^+^ EVs, which remain to be investigated in future studies.

We further assessed the ability of PentraSorb, which is clinically used to deplete CRP from human plasma in myocardial infarction^32,50,51^, to bind CRP-carrying EVs. CRP^+^ EVs were undetectable after adsorbent treatment of septic plasma, whereas total EV counts remained largely unaffected, indicating a detachment of CRP from the EV surface by adsorbent treatment rather than a direct depletion of CRP^+^ EVs. Along with the depletion of EV-bound CRP, PentraSorb treatment abrogated EV-induced IL-8 secretion from human monocytes, supporting the immune modulatory capacity of EV-bound CRP.

In conclusion, our study demonstrates that platelet-derived circulating EVs are associated with CRP in sepsis. By inducing the release of IL-8 from monocytes, CRP^+^ EVs may contribute to local immune cell chemotaxis. Finally, we provide evidence that CRP can be detached from circulating EVs using PentraSorb.

Beyond the potential relevance of CRP^+^ EVs in immune modulation^13,14^, our findings highlight a general role of EVs in binding of plasma proteins to stabilize or solubilize them for transport, or in changing the bioactivity of their cargo by mediating conformational changes.

## Methods

### Human whole blood and plasma

Whole blood was drawn from healthy volunteer individuals into vacutainer tubes (Vacuette, Greiner Bio-One, Kremsmuenster, Austria) containing sodium citrate or EDTA, as stated for the individual experiments. Blood collection was approved by the Ethical Review Board of Danube University Krems. Whole blood from sepsis patients was obtained from the University Clinic St. Pölten, Austria, as approved by the Lower Austrian Ethics Committee (GS4-EK-3/082–2012) and in accordance with the Declaration of Helsinki. Written informed consent was obtained from all donors or their legal guardians.

Platelet poor plasma was generated by immediate centrifugation of whole blood anticoagulated with citrate at 2000×g (15 min, 4°C), and was stored at − 80°C until further use.

### Antibodies and cell culture reagents

Antibodies and fluorochrome conjugates used for flow cytometry and Western Blotting are specified in Table 1. Phosphate buffered saline (PBS) without calcium and magnesium was obtained from Life Technologies (Paisley, UK). EDTA was purchased from VWR (Radnor, PA). RPMI-1640 medium was supplemented with 20 mM 4-(2-hydroxyethyl)-1-piperazineethanesulfonic acid (HEPES), 100 µg/mL streptomycin, 100 IU/mL penicillin (all from Sigma Aldrich, St. Louis, MO). Human AB serum (Sigma Aldrich) was centrifuged at 20,000×*g* for 30 min at 4°C and sterile filtered (0.2 µm filter) to deplete EVs prior to use. Trypan blue was obtained from Sigma Aldrich.

**Table 1:** Fluorochrome-labeled antibodies used for flow cytometry and Western blotting.

Antigen	Source	Clone	Marker for	Fluorochrome	Abbreviation	Dilution	Supplier	Catalog number
**Flow cytometry**								
CD14	Mouse	RMO52	Monocytes	Phycoerythrin	PE	1:10	Beckman Coulter	A07764
CD14	Mouse	RMO52	Monocytes	Allophycocyanin Alexa Fluor 750	APC AF750	1:50	Beckman Coulter	B92421
CD41	Mouse	P2	Platelets	Phycoerythrin Cyanin 7	PC7	1:20	Beckman Coulter	6607115
CD45	Mouse	J33	Leukocytes	Pacific Blue	PB	1:10	Beckman Coulter	A74763
CD235	Mouse	HIR2 (GA-R2)	Red blood cells	Allophycocyanin	APC	1:50	eBioscience	17–9987-42
CRP	Goat	Not applicable	C-reactive protein	Fluorescein Isothiocyanate	FITC	1:100	Abcam	ab34659
Annexin5	Not applicable	Not applicable	Phosphatidylserine	Phycoerythrin	PE	1:40	BD Biosciences	556421
**Western blotting**								
CRP	Mouse	CRP-8	C-reactive protein	–	–	1:30,400	Sigma Aldrich	C1688

### Quantification of protein, CRP, and IL-8

Protein was quantified with the DC Protein Assay (Bio-Rad, Hercules, CA). Plasma CRP as well as IL-8 levels in cell culture supernatants were determined by enzyme-linked immunosorbent assay (ELISA; both from R&D Systems, Minneapolis, MN) according to the instructions of the manufacturer.

### Characterization of extracellular vesicles in plasma samples using flow cytometry

Platelet poor plasma from sepsis patients (n = 30) was diluted 500-fold in Anx5 binding buffer (BD Biosciences, Bedford, MA), and EVs were stained with PE-conjugated Anx5 as marker for EVs exposing phosphatidylserine and with a FITC-conjugated anti-CRP antibody for 15 min in the dark at room temperature (RT). Plasma from healthy individuals (n = 5) was diluted 50-fold in Anx5 binding buffer and stained under identical conditions. Additionally, to determine the cellular origin of EVs, staining with Anx5 and anti-CRP antibody was combined with an APC-conjugated anti-CD235a antibody (eBioscience, San Diego, CA, USA) as red blood cell marker, a PC7-conjugated anti-CD41 antibody as platelet marker, an APC AF750-conjugated anti-CD14 antibody as monocyte marker, and a PB-conjugated anti-CD45 antibody for leukocyte marker (all from Beckman Coulter, Brea, CA). EVs were characterized using a Gallios flow cytometer (Beckman Coulter) equipped with 405 nm, 488 nm, and 638 nm lasers after calibration with fluorescent-green silica beads (1 µm, 0.5 µm, 0.3 µm; excitation/emission 485/510 nm; Kisker Biotech, Steinfurt, Germany). The triggering signal was set on the forward scatter/size, and the EV gate was defined as previously described^43^ and as shown in Supplementary Fig. S1a. The lower size limit of detection was 250 nm^35^. To confirm the presence of intact EVs, detergent lysis with 0.25% Triton-X 100 (Sigma Aldrich) was performed. Data were acquired for 3 min at a flow rate of 10 μL/min and analyzed using the Kaluza software, version 1.3 (Beckman Coulter). All fluorochrome-labeled antibodies used for flow cytometry are specified in Table 1 and were centrifuged at 17,000×*g* for 10 min before use. Buffer controls, fluorochrome-labeled reagent controls (to exclude unspecific binding, unfiltered Anx5 binding buffer, which contains non-defined particles, was stained with Anx5-PE or anti-CRP-FITC), as well as single antibody stainings are shown in Supplementary Fig. S1b,c.

### Adsorbent treatment of plasma from sepsis patients

PentraSorb CRP (Pentracor, Hennigsdorf, Germany), further designated as PentraSorb, is clinically approved for therapeutic apheresis to deplete CRP from the circulation. The adsorbent is composed of agarose beads, which are functionalized with phosphocholine derivatives^33^. To assess the depletion of CRP and CRP^+^ EVs by PentraSorb, plasma samples from sepsis patients containing > 50% CRP^+^ EVs were incubated with 10 vol% of adsorbent for 30 and 60 min at 37°C with gentle agitation. Thereafter, adsorbent beads were pelleted by centrifugation at 2000×g for 5 min. Total EV counts, CRP^+^ EVs, as well as plasma CRP levels were determined by flow cytometry and ELISA as described above. Untreated plasma and plasma treated with non-functionalized agarose beads (adsorbent matrix) under otherwise identical conditions served as controls.

### Isolation of primary human monocytes

Freshly drawn human whole blood anticoagulated with EDTA was diluted 1:2 in PBS containing 5 mM EDTA, and peripheral blood mononuclear cells (PBMCs) were enriched by density gradient centrifugation on Ficoll-Paque PLUS (GE Healthcare, Uppsala, Sweden) as previously described^52^. Monocytes were isolated from PBMCs by negative depletion of non-monocytes (Pan Monocyte Isolation Kit, Miltenyi Biotec, Bergisch Gladbach, Germany) as previously described^42^. Their purity was determined by cell counting (Sysmex KX-21N, Sysmex, Neumuenster, Germany) as well as by flow cytometry after staining with anti-CD45-PB and anti-CD14-PE as monocyte markers as well as anti-CD41-PC7 for platelet origin. Monocyte purity was calculated as ratio of CD14^+^CD45^+^ cells (monocytes) to all CD45^+^ cells. Monocyte viability was assessed by trypan blue exclusion. Monocytes were kept in RPMI medium supplemented with 10% EV-depleted AB serum in suspension culture plates (Greiner Bio-One) overnight prior to further use.

### Enrichment of extracellular vesicles

EVs were enriched from aliquots of 500 µL platelet poor plasma from either sepsis patients (n = 7) or healthy individuals (n = 4). Plasma samples were centrifuged at 20,000×*g* (30 min, 4°C; Sorvall Evolution RC centrifuge, SS34 fixed-angle rotor, Thermo Fisher Scientific, Waltham, MA). Supernatants were collected, and the pellets were washed with 500 µL PBS, re-centrifuged, and re-suspended in 10 µL PBS.

### Detection of CRP in extracellular vesicle fractions using western blotting

Following the enrichment of EVs from plasma samples (see above), EV-depleted supernatants and EV pellets (15 µg protein each), as well as native human CRP (0.5, 0.1, 0.05 µg protein; Bio-Rad) were separated by sodium dodecyl sulfate polyacrylamide gel electrophoresis (SDS-PAGE) under reducing conditions (any kDa gels, Mini-PROTEAN TGX, Bio-Rad). Proteins were blotted onto nitrocellulose membranes (Bio-Rad), probed for CRP with an anti-CRP antibody as specified in Table 1, and detected using the Western Breeze chemiluminescent kit (Invitrogen, Carlsbad, CA).

### Stimulation of monocytes with extracellular vesicles

Primary human monocytes (see above) were seeded into 96-well suspension plates (Greiner Bio-One) at a density of 5 × 10^4^ cells in 200 µL RPMI medium supplemented with 10% EV-depleted AB serum. They were stimulated with EVs isolated from 500 µL of plasma from sepsis patients (10,557–19,709 CRP^+^ EVs/µL; 59.0 ± 7.4% CRP^+^ EVs; range: 48.1–64.9%; n = 7), which was either pre-treated with 10 vol% of PentraSorb for 30 min or left untreated. EVs isolated from plasma from healthy individuals (n = 4) served as negative control. Following monocyte stimulation for 8 h at 37°C in 5% CO_2_ in humidified atmosphere, cell culture supernatants were collected by centrifugation at 600 g (5 min, 4°C), and stored at − 80°C until further analysis.

### Statistical analysis

Statistical analysis was performed using GraphPad Prism version 7.02 (La Jolla, CA). Data were tested for normality using the Shapiro–Wilk test. To compare normally distributed data, an unpaired t-test (with or without Welch’s correction) was used, while a Mann–Whitney test (unpaired) was used for non-normally distributed data (all two-tailed). For multiple comparisons of data, two-way ANOVA followed by Bonferroni’s comparisons test was applied. Spearman test was used to calculate correlations between soluble CRP and CRP^+^ EV counts. Data are presented as mean ± standard deviation (SD). Significance was accepted at p < 0.05.

## Supplementary Information

Supplementary Information.

## Data Availability

Authors provide adequate assurance that they can comply with the publication’s requirements for sharing material.
