# Supplementary material for: Extracellular vesicles are associated with C-reactive protein in sepsis
Source: Sci Rep. 2021 Mar 26;11:6996. doi: 10.1038/s41598-021-86489-4 (PMC7997920; doi:10.1038/s41598-021-86489-4)
Supplement: Supplementary file 1 — Supplementary Information. [file 41598_2021_86489_MOESM1_ESM.pdf]

Supporting Information

Extracellular vesicles are associated with C-reactive protein in sepsis

Birgit Fendl[1], René Weiss[1], Tanja Eichhorn[1], Ingrid Linsberger[1], Taras Afonyushkin[2], Florian Puhm[2], Christoph J. Binder[2], Michael B. Fischer[3], Viktoria Weber[1*]

[1]Christian Doppler Laboratory for Innovative Therapy Approaches in Sepsis, Department for Biomedical Research, Danube University Krems, Krems, Austria;
[2]Department of Laboratory Medicine, Medical University of Vienna, Vienna, Austria;
[3]Center for Experimental Medicine, Department for Biomedical Research, Danube University Krems, Krems, Austria

*To whom correspondence should be addressed: Prof. Viktoria Weber, Danube University Krems, Dr.-Karl-Dorrek-Strasse 30, 3500 Krems, Austria; viktoria.weber@donau-uni.ac.at; phone +43 2732 893 2632.

Content: Supplementary Figure S1, Supplementary Figure S2, Supplementary Figure S3

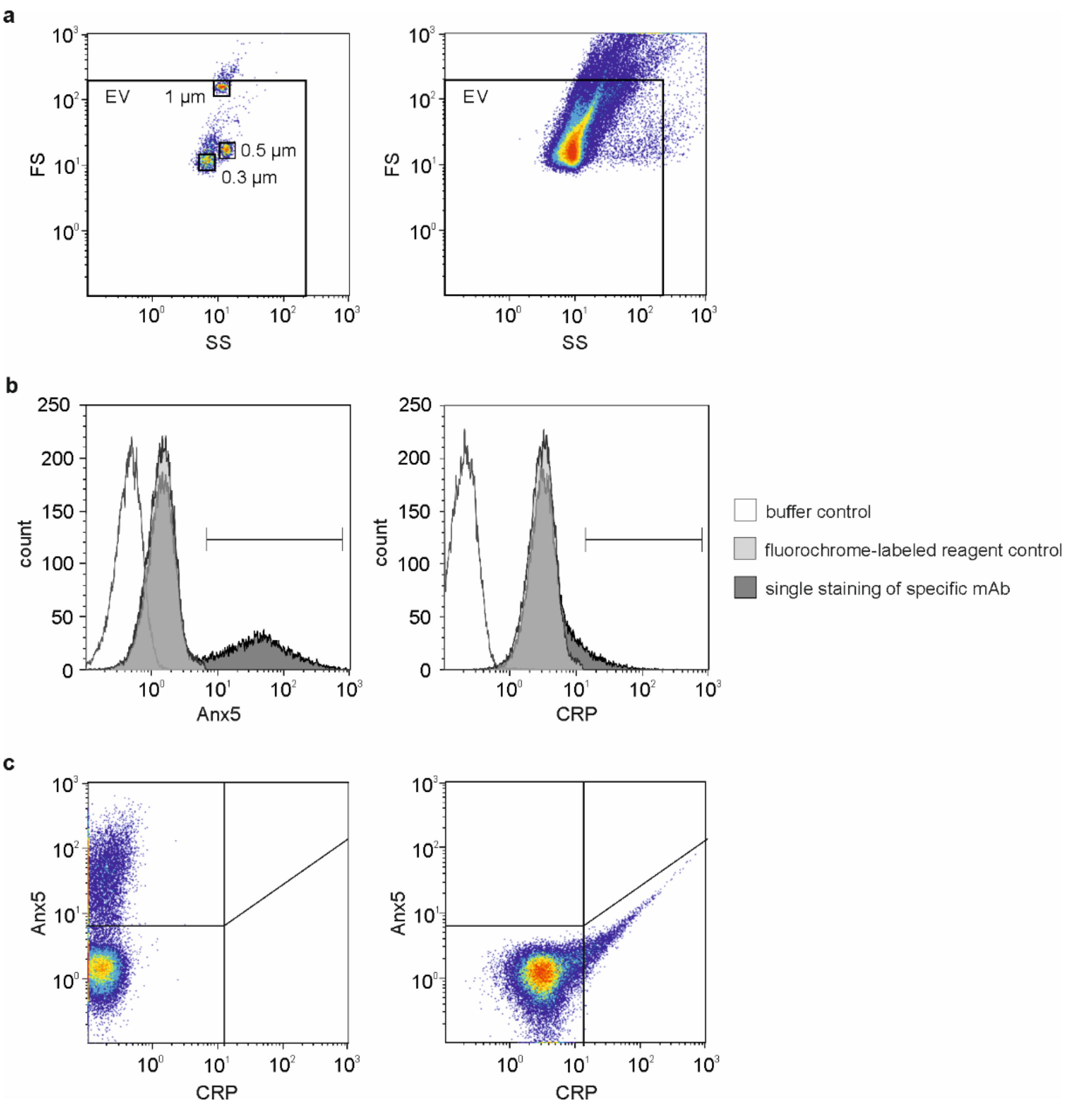

Supplementary Figure S1. Flow cytometric characterization of EVs: calibration and controls. (a) The flow cytometer (Gallios, Beckman Coulter, Brea, CA) was calibrated using fluorescent-green silica beads (1 µm, 0.5 µm, 0.3 µm, Kisker Biotech, Steinfurt, Germany) and the EV gate was set at the 1 µm bead cloud as described in the main manuscript (**left**); Representative side scatter (SS) *vs.* forward scatter (FS) plot of the EV cloud detected in plasma from sepsis patients (**right**). **(b)** Buffer controls and fluorochrome-labeled reagent controls were used to identify Anx5 positive events (**left**) and CRP positive events (**right**). **(c)** Single staining of septic plasma sample with Anx5 **(left)** and CRP **(right)**.

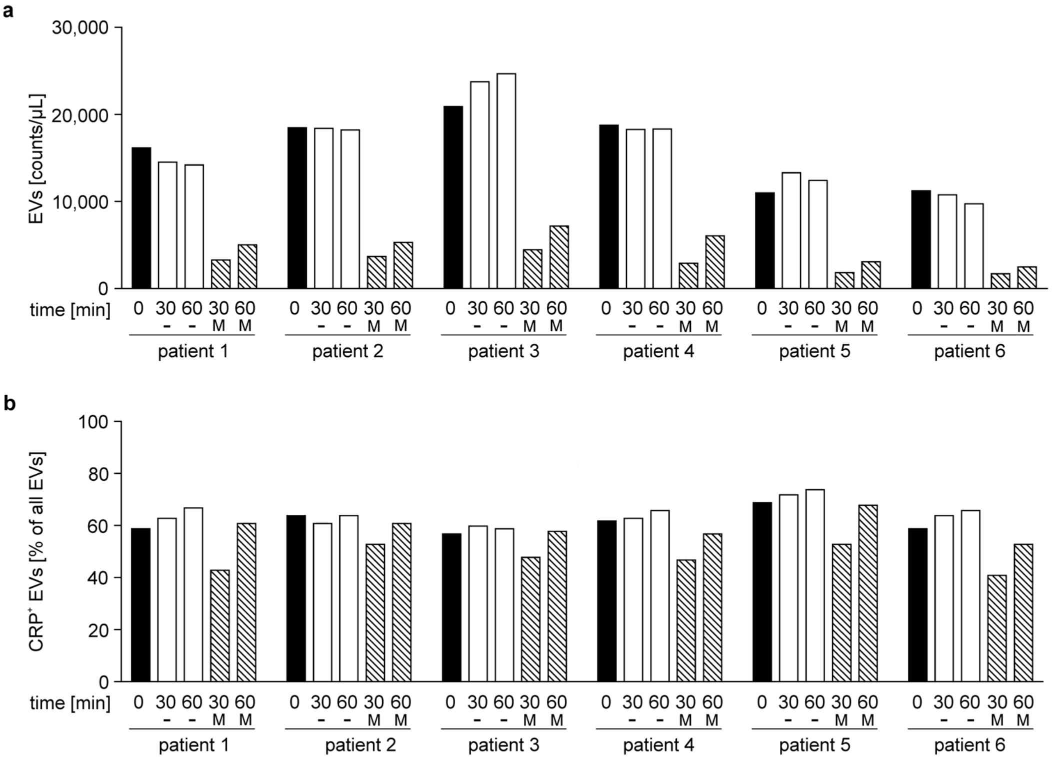

Supplementary Figure S2. Treatment of plasma from sepsis patients with non-functionalized agarose. In addition to treatment with PentraSorb (main manuscript, Fig. 2), plasma from sepsis patients (6 patients; n=1 for each patient) was incubated with 10 vol% non-functionalized agarose (adsorbent matrix, M) or left untreated (-) as described in the Methods section. EV counts **(a)**, CRP-carrying (CRP^+) EVs **(b)**, as well as plasma CRP were quantified at baseline (0 min), and after 30 and 60 min.

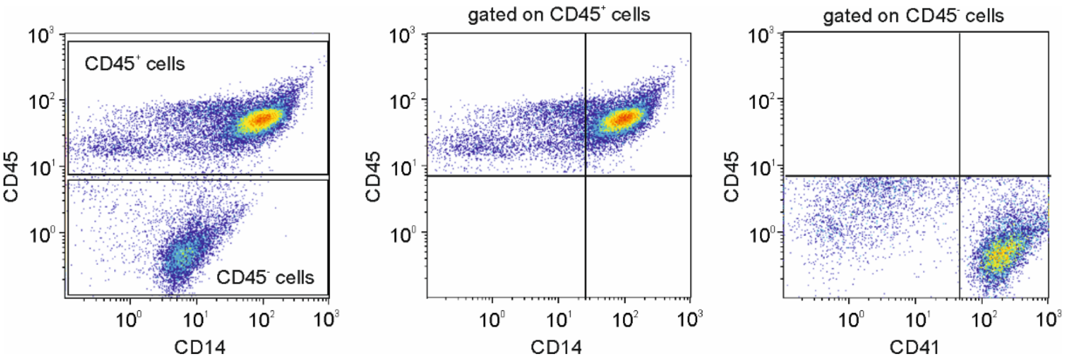

Supplementary Figure S3. Purity of isolated monocytes. Monocytes were isolated from peripheral blood mononuclear cells by negative depletion using the Pan Monocyte Isolation Kit as described in the Methods section. Cells were stained with anti-CD14-PE, anti-CD45-PB, as well as anti-CD41-PC7. Monocytes were identified as $CD45^{+}CD14^{+}$ cells, and their purity was calculated in relation to all leukocytes ($CD45^{+}$ cells; **left and middle**). Residual platelets were identified via their expression of CD41 (**right**).
